# Long‐lived marine species may be resilient to environmental variability through a temporal portfolio effect

**DOI:** 10.1002/ece3.6378

**Published:** 2020-05-25

**Authors:** Jacek Maselko, Kimberly R. Andrews, Paul A. Hohenlohe

**Affiliations:** ^1^ College of Science, Bioinformatics and Computational Biology Program University of Idaho Moscow ID USA; ^2^ Alaska Fisheries Science Center NOAA National Marine Fisheries Service Juneau AK USA; ^3^ Institute for Bioinformatics and Evolutionary Studies University of Idaho Moscow ID USA; ^4^ Department of Biological Sciences Institute for Bioinformatics and Evolutionary Studies University of Idaho Moscow ID USA

**Keywords:** adaptive variation, genotype–environment association, portfolio effect, RADseq, selection, selective sieve

## Abstract

Maintenance of genetic variation may provide resilience of populations to natural environmental variability. We used Pacific ocean perch (POP; *Sebastes alutus*) to test for the maintenance of adaptive variation across overlapping generations. POP are a long‐lived species characterized by widespread larval dispersal in their first year and a longevity of over 100 years. In order to understand how early marine dispersal affects POP survival and population structure, we used restriction site‐associated DNA sequencing (RADseq) to obtain 11,146 single‐nucleotide polymorphisms (SNPs) from 401 young‐of‐the‐year (YOY) POP collected during surveys conducted in 2014 (19 stations) and 2015 (4 stations) in the eastern Gulf of Alaska. Population clustering analysis showed that the POP samples represented four distinct ancestral populations mixed throughout the sampling area. Based on prior work on larval dispersal of POP, these larvae are most likely from distinct parturition locations that are mixing during their pelagic dispersal life stage. Latent factor mixed models revealed that POP larvae face significant selection during their first year at sea, which is specific to the year of their birth. Thus each adult cohort's genetic composition is heavily influenced by the environmental conditions experienced during their first year at sea. Long‐lived species relying on broadcast spawning strategies may therefore be uniquely resilient to environmental variability by maintaining a portfolio of cohort‐specific adaptive genotypes, and age truncation due to overfishing of older cohorts may have detrimental effect on the population viability.

## Graphical abstract

We found that marine fish larvae and young‐of‐the year Pacific ocean perch (*Sebastes alutus*) experience strong selection acting during their pelagic stage. This selection is dependent on the environmental conditions encountered during their first year of life. We present evidence of fluctuating selection acting on Pacific ocean perch young‐of‐the year life stage resulting in cohort‐specific genetic selection patterns.

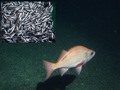

## 1 INTRODUCTION

Understanding the resilience of biological marine resources to changing oceanographic conditions is central to ecosystem‐based fisheries management and the implementation of adaptive sustainable harvest strategies (Levin & Möllmann, 2015; Link, 2002). The ability of populations to respond to disturbances in their habitat is in part determined by the genetic variation present in the population (Hoffmann & Sgro, 2011; Parker et al., 2000). This genetic variation, exhibited by a portfolio of available gene variants, allows for a quick response if selectively advantageous variants are already present in the population (Pacifici et al., 2015; Sunday, Crim, Harley, & Hart, 2011). Understanding the response of marine populations to environmental perturbations will allow us to readily assess the resilience or vulnerability of these populations and species as a whole.

Measuring differential survival between subpopulations reveals how environmental conditions can influence the overall productivity of exploited populations. For example, Schindler et al. (2010) demonstrated that environmental conditions favored the production of discrete salmon populations residing in Bristol Bay, Alaska. In a follow‐up study, Larson et al. (2019) showed river ecotypes of sockeye salmon displaying higher genetic diversity than those of beach spawners. They attributed it to a more homogeneous beach habitat than that in streams as well as higher stream spawning densities. The maintenance of genetic variation may therefore be a key aspect of resilience of populations to natural environmental variability.

The idea of a temporal portfolio effect, in which adaptive variation is maintained by overlapping generations in a temporally variable environment, has been studied in general (Ellner & Hairston, 1994). For example, many freshwater zooplankton taxa have relatively short‐lived adults that may be subject to strong selection, but eggs can remain viable for decades in sediment, resulting in persistent egg banks that are relatively buffered from environmental variation (Brendonck & De Meester, 2003). We hypothesize that a similar effect may occur in marine fish species with highly dispersive larvae and long‐lived, relatively sedentary adults. The genetic composition of each recruitment cohort may reflect relatively strong selection during the larval stage, while the adult population would maintain genetic variation reflecting multiple cohorts. Here, we test this hypothesis using genomic methods for detecting population structure and adaptive loci.

Genomic data allow us to scan for individual and population‐level differences across the whole genome, and genomics is becoming integral in answering a wide array of previously unresolved questions in conservation biology with numerous applications in fisheries (Barrio et al., 2016; Jasonowicz, Goetz, Goetz, & Nichols, 2016; Kumar & Kocour, 2017; Valenzuela‐Quiñonez, 2016; Wenne et al., 2007). It is now possible to estimate, with a high level of precision and certainty, the demographic structure of fish populations at small spatiotemporal scales and to identify local adaptation from genomic data (Barrio et al., 2016; Catchen et al., 2017; McKinney, Larson, Seeb, & Seeb, 2017; Wang & Höök, 2009). RADseq approaches have been extensively used to describe various biological and ecological phenomena, such as phylogeography, population differentiation and structure, population and individual admixture (composition of lineages), genetic diversity, and outlier loci detection, among others (Alexander, Novembre, & Lange, 2009; Andrews, Good, Miller, Luikart, & Hohenlohe, 2016; Narum, Buerkle, Davey, Miller, & Hohenlohe, 2013).

**FIGURE 1:**
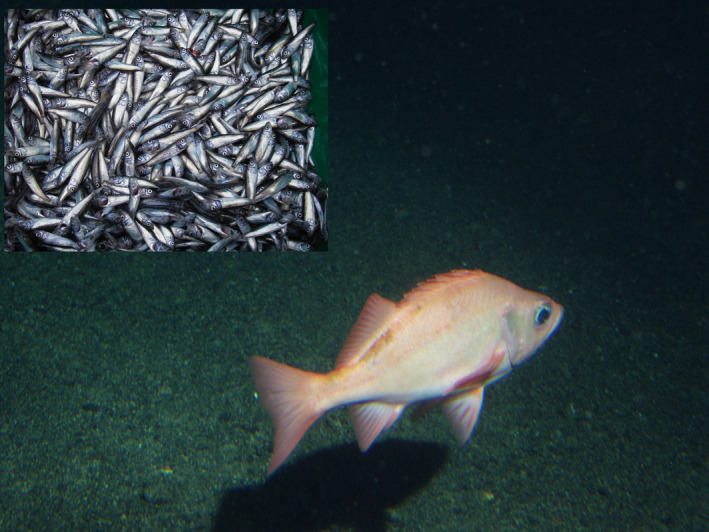
The Pacific ocean perch (*Sebastes alutus*) is a long‐lived species characterized by widespread dispersal during their first year and a longevity of over 100 years. Main photo is of the adult semidemersal Pacific ocean perch, while the inset is of the young of the year, pelagic life stage, as collected for this study (photo by NOAA)

Our model species, the Pacific ocean perch (POP; *Sebastes alutus*, Figure 1), is a long‐lived species with its oldest individuals being over 100 years old (Conrath & Knoth, 2013). POP are the most abundant and economically important rockfish species in the Gulf of Alaska (Conrath & Knoth, 2013) with landings in excess of 55,000 tons in 2017 (NOAA, 2019). The fishery is managed using an age‐structured model where the vital population rates are derived from the abundances of different ages in the catch (Hulson, Hanselman, Lunsford, & Fissel, 2017; Megrey, 1988), but the relationship between the abundance of spawning fish and their offspring cohort is highly variable and unpredictable. This extreme annual fluctuation in success and failures of various year classes has been noted as a characteristic of this and many other commercially exploited species (Carlson & Haight, 1976; Westrheim, 1958).

Pacific ocean perch in the Gulf of Alaska live on the upper slope of the continental shelf. They spawn from September through November, with parturition occurring in April through May the following year, when larvae rise from demersal spawning habitats on the continental shelf break (150 – 400m depth) to surface waters. They then become part of the ichthyoplankton and within a few weeks metamorphose to a young‐of‐the‐year form (YOY). They are carried in the surface waters by currents and settle out of the water column in nearshore rocky habitat by the end of their first year (Carlson & Haight, 1976; Major & Shippen, 1970). During their shoreward movement, larvae grow rapidly and allocate significant amounts of energy to creating lipid tissue. This lipid tissue is apparently lost during settlement (Moss et al., 2016), suggesting energy acquisition and growth are important determinants of settlement success (Hoey & McCormick, 2004). They remain in the nearshore habitat for the next few years until they join the discrete adult schools residing on the continental shelf and slope (Love, Yoklavich, & Thorsteinson, 2002). They reach sexual maturity at eight to ten years of age and repeatedly spawn until their hundredth year or longer (Hulson et al., 2017). These adult schools are genetically differentiated, and the degree of their differentiation (*F*_ST_) is correlated to the geographic distance between them (Palof, Heifetz, & Gharrett, 2011).

Larval dispersal pathways in POP may be highly variable from year to year since they mostly depend on ocean currents in a given year (Mundy et al. 2010). An important prediction of ocean current and dispersal models (Stockhausen, 2009; Stockhausen & Hermann, 2007) is that in each year, the larvae at a given pelagic location are comprised of mixtures of individuals from different spawning locations indicating a high degree of mixing among them. However, population genetic studies of young of the year and adults indicate there is limited mixing among subpopulations (Kamin, Palof, Heifetz, & Gharrett, 2014; Palof et al., 2011). The Kamin et al. (2014) follow‐up study examined the YOY POP catches corresponding to locations near the adults caught by Palof et al. (2011). Their work showed that the collections of YOY POP were most related to the linearly closest adult populations. Either widely dispersed juveniles are able to return to their natal areas, or survival is maximized among locally retained larva, possibly due to local adaptation.

Here, we test whether POP larvae exhibit signatures of selection that could allow for the maintenance of a portfolio of adaptive variation in the multicohort adult population. We examined YOY POP collected from the eastern Gulf of Alaska during 2 years (2014 and 2015), when the oceanographic conditions were drastically different, with 2014 being an average temperature year, and 2015 being anomalously warm which is expected to have a negative impact on the fish (Cavole et al., 2016; Gentemann, Fewings, & García‐Reyes, 2017; Jones et al., 2018). We evaluated the potential for differences in selection strength for YOY POP across years by testing whether the fish differed in physiological conditions in 2014 and 2015, measured as a body condition index based on weight–length relationships, and total lipid content. We then used genotype–environment association (GEA) tests with RADseq genomic data to test for differences in selection acting on the genome to favor different phenotypes between the two years. Finally, we identified candidate biological pathways on which selection was acting in the two different YOY cohorts. We predicted that the strength of selection would be higher in 2015 than 2014 due to the unusually high 2015 sea temperatures, resulting in poor body condition and a greater number of SNPs associated with environmental variables and physiological condition in the 2015 dataset. These findings may explain the difference in recruitment for the 2014 and 2015 cohorts as estimated in the 2017 stock assessment (Hulson et al., 2017).

## 2 MATERIALS AND METHODS

### 2.1 Sample collection and processing

**FIGURE 2:**
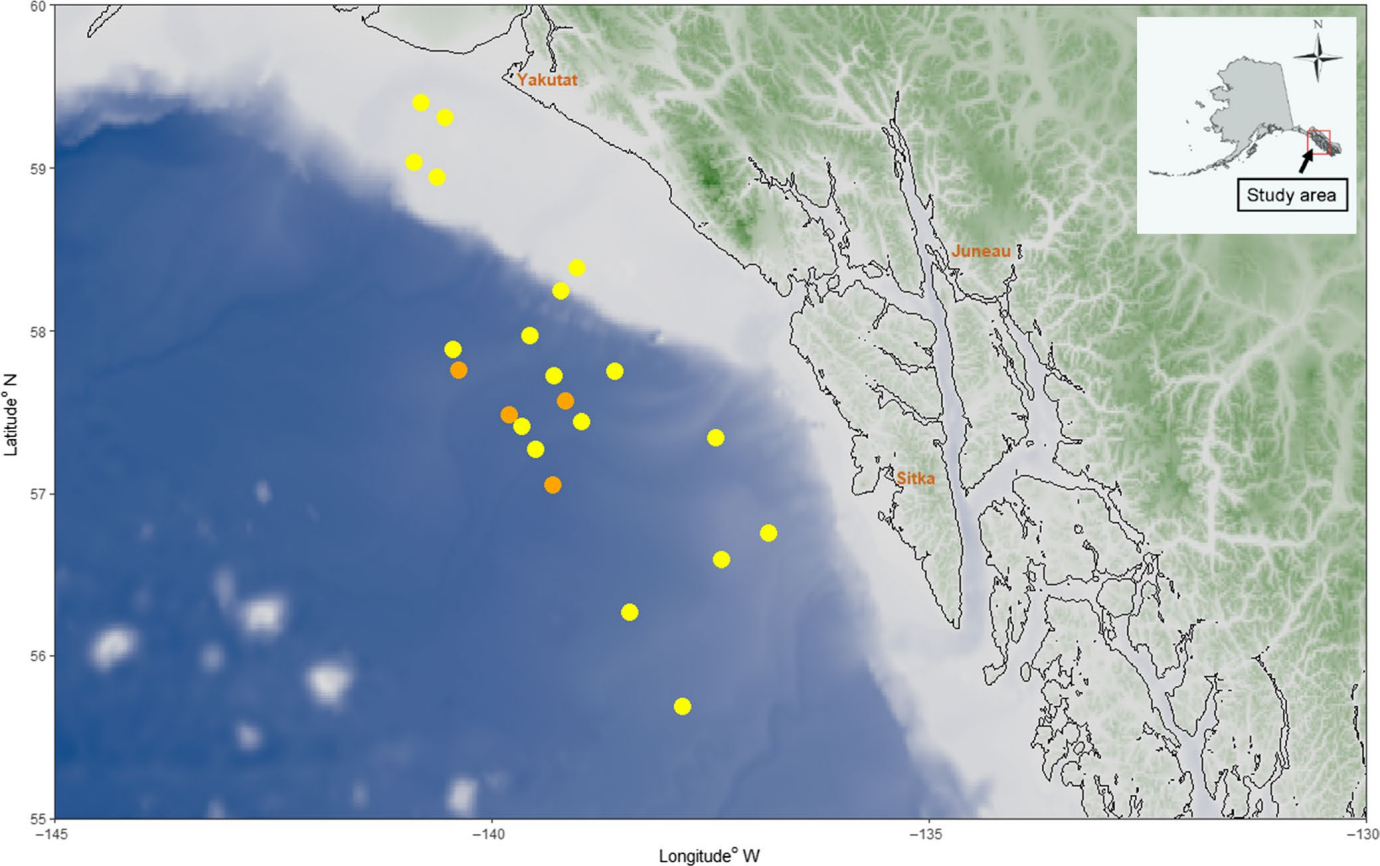
Locations of the 2014 (yellow) and 2015 (orange) collection of the young‐of‐the‐year Pacific ocean perch

Young‐of‐the‐year (YOY) POP were collected during NOAA oceanographic surveys in the summer of 2014 (July 8–August 14) and 2015 (July 13–July 22) (Figure 2). POP larvae and YOY were distinguished from congenerics (Kendall, Kondzela, Li, Clausen, & Gharrett, 2007) using diagnostic SNPs (Garvin et al., 2011) prior to inclusion in this study. The resulting sample size of identified YOY POP was 399 fish in 2014 and 108 in 2015.

Fish length, weight, and lipid content for each identified POP fish were measured at Auke Bay Laboratories in Juneau, AK. Lipid content was extracted using the Folch method (Folch, Lees, & Sloane Stanley, 1957) and quantified using the colorimetric sulpho‐phospho‐vanillin (SPV) method (Chabrol & Charonnet, 1937). Condition index was calculated as the residual value from a log(weight)~log(length) linear regression. This index accounts for the different lengths due to age of the YOY fish where a positive residual indicates better body condition than expected (Froese, 2006). Finally, for DNA analysis, a small tissue plug was extracted through an incision in the abdominal wall that included the heart tissue and stored in 95% ethanol.

### 2.2 Molecular analysis

DNA was extracted from the muscle plug from the 515 individual fish into 96‐well plates with the QIAGEN DNeasy Blood and Tissue Kits as described by the manufacturer (Qiagen, Inc.). Individual samples were distributed among the six total plates to account for any plate bias. In brief, small pieces of tissue (~20 mg) were excised from each muscle plug. The tissue pieces were digested in a proteinase solution for at least 3 hr at 55°C. Protease digestions were performed in 96‐well plates. After digestion, the samples were purified with either QIAxtractor or Corbett X‐tractor robot producing eluted DNA which was stored at −20°C.

RADseq library preparation was done for all 513 samples including eight samples that were replicates, according to Ali et al. (2016) and refined by Andrews et al. (2018) using the Sbf1 restriction enzyme, which cuts at an eight‐base recognition site. Custom eight‐base biotinylated barcodes were ligated to the cut site allowing multiplexing of groups of 96 samples. The multiplexed samples were then sheared to 400 bp using Covaris M220 sonicator. This was followed by a streptavidin bead assay to exclude sheared fragments that did not include the biotinylated barcodes. Illumina's NEBNext ultra DNA library prep kit was then used to add Illumina adapters with indexes unique to each of the multiplexed groups of 96 samples to allow further pooling and Illumina sequencing compatibility. 150‐bp paired‐end sequencing was done on two lanes at the Berkeley Genomics Center Laboratory (https://qb3.berkeley.edu/gsl/) using Illumina HiSeq 4000.

### 2.3 Sequencing and data processing

We followed the bioinformatic pipeline described in Andrews et al. (2018) (Figure 1), with slight modification for STACKS version 2.0 (Catchen, Hohenlohe, Bassham, Amores, & Cresko, 2013). Briefly, a custom PERL script was used to flip the raw reads so that each 140 bp read was aligned starting with the barcode, and the Sbf1 cut site sequence. STACKS 2.0 (Catchen et al., 2013) program *process_radtags* was used to demultiplex the raw reads followed by program *clone_filter* to remove PCR duplicates. BOWTIE2 version 2.3.4.3 (Langmead & Salzberg, 2012) was used to align the sequences to *Sebastes nigrocinctus* reference genomes downloaded from the ncbi database (https://www.ncbi.nlm.nih.gov/genome/14568). The *S. nigrocinctus* aligned reads were then processed using the refmap.pl pipeline in STACKS 2.0. Filtering of the final set of SNPs was done using POPULATIONS module in STACKS 2.0 with the minimum percent of individuals genotyped at a locus in a population set at 10% and the minimum global minor allele frequency of SNPs set at 0.1. We used this stringent MAF filter to minimize noise due to low‐frequency alleles, which are less informative about admixture (Linck & Battey, 2019). Subsequent analysis was conducted using R statistical software (R Core Team, 2016) using data in *genepop* format exported from POPULATIONS module.

CLUSTER analysis was conducted using package *adegenet* (Jombart, Devillard, & Balloux, 2010) and *poppr* (Kamvar, Brooks, & Grünwald, 2015; Kamvar, Tabima, & Grünwald, 2014) using all samples, including the seven remaining replicate pairs (one replicate did not pass the missing data filter) to select the optimal set of filters for removing individuals and loci based on the level of missing data. These filter settings were varied until the CLUSTER plot showed the paired replicates to be most closely related. This resulted in removal of loci which were absent in at least 15% of individuals and genotypes having more than 20% of total identified loci missing. For subsequent analyses, only one from each pair of replicate samples with the most loci was retained. We used the R package *s equoia* (Huisman, 2017) to identify related individuals, up to half‐siblings; this program is specifically designed to use large SNP datasets and does not require a parent to be present in the sample. This was done for each of the two cohorts in order to make sure no related individuals were included in the genome–environment association (GEA) tests.

We estimated the number of ancestral populations represented in the sample using the *LEA* R package (Frichot & Francois, 2015). The analysis employed population clustering analysis with sparse non‐negative matrix factorization optimization (sNMF) (Frichot, Mathieu, Trouillon, Bouchard, & François, 2014) to estimate number of ancestral populations represented in the sample. The number of populations was determined from the cross‐entropy criteria and Cattell's rule (Cattell, 1966) from the sNMF output. We favored the sNMF algorithm because it is robust to departures from Hardy–Weinberg equilibrium as compared to Bayesian and maximum‐likelihood approaches (Frichot et al., 2014). We also compared the sNMF results to STRUCTURE 2.3.4 (Pritchard, Stephens, & Donnelly, 2000) derived population clustering.

We examined whether selection pressure is consistent from year to year by testing for a difference in the number of private alleles or homozygous loci in each year. Under stochastic processes other than selection, such as genetic drift, we would expect a random number of private alleles distributed throughout the genome in each population and year. However, if one cohort experienced strong directional selection, we should observe the advantageous alleles only, whereas both alleles are expected in the absence of selection. The number of private alleles that were only found in 2014 but not in 2015 was therefore quantified specifically to each sNMF‐derived population and across all SNPs. If the specific private alleles were conserved among these sNMF‐derived populations, it would suggest that this loss of alleles in 2015 was not due to a sampling effect only.

We also needed to account for the large discrepancy in sample sizes between 2014 and 2015 which was due to large differences in all forage fish abundances resulting in poor 2015 catches. To compute whether the number of private alleles was significantly different between the two years and not just due to a smaller sample size, we needed to account for the difference in sample sizes. We wrote a custom permutation routine in R to create a null distribution of the expected number of lost alleles for a given sample size by selecting without replacement from the combined sample distribution (Efron & Tibshirani, 1994) (see Algorithm in Appendix S1). The significance (*p* = .05) was then based on where the observed number of private alleles lies in the null distribution.

Latent factors mixed model (LFMM) algorithm in R package *LEA* (Frichot & Francois,^2014^) was conducted to identify loci influenced by selection. We used LFMM algorithm for genome–environment association study in order to account for population structure in the dataset. Unlike OutFLANK or PCAdapt, LFMM algorithm simultaneously estimates the latent factors (demography) with environmental response variables. This makes it more robust to the presence of confounding factors, and it does not require the creation null *F*_ST_ distribution. We conducted the LFMM analysis separately for each environmental and phenotypic variable for *K* values of 3–5, with 10 repetitions, 20,000 iterations, and burnin of 5,000. For subsequent analysis, we imputed any remaining missing data (3.5% in 2014 and 4.1% in 2015). The missing genotypes were imputed using the random forest algorithm in the R packages *randomForestSRC* and *radiator* (Gosselin, 2018). We used the R package *hierfstat* (Goudet, 2005) to estimate pairwise *F*_ST_ according to Nei (1987). Significance of *F*_ST_ was calculated through 1,000 permutations of population indices. PCA analysis was conducted using the dudi.pca routine in *ade4* R package (Dray & Dufour, 2007). Environmental variables included in the genome–environment association (GEA) were obtained from the cruise data collections and consisted of sample date and latitude, seawater temperature, and chlorophyll concentration. Phenotypic metrics included in the GEA were collected back at Auke Bay Laboratory, including percent lipid content and condition index. This analysis was done for 2014 and 2015 data separately with four latent factors to account for population structure while testing for genome–environment association. This was followed by nucleotide BLAST (https://blast.ncbi.nlm.nih.gov/Blast.cgi) search of nucleotide sequences and their corresponding protein‐coding gene regions where selection may be occurring. Loci annotation and BLAST searches of the associated 140 bp sequences were accepted when below the nucleotide and protein e‐value threshold of 1 × 10^−10^. BLAST e‐value score is the probability that the similarity is due to chance.

The gene ontology (GO) enrichment analysis was used to determine whether the groups of genes associated with each of the environmental variables were enriched for certain biological processes. This analysis was done by querying the http://geneontology.org database using zebra fish (*Danio rerio*) as a reference organism, and the alpha level was set at *p* = .05 with no multiple test correction applied. Subsequently, the www.biocyc.org and www.informatics.jax.org were queried to determine general biological functions of the gene aggregates.

## 3 RESULTS

### 3.1 Bioinformatics and population grouping

The total number of raw Illumina sequencing reads for the six plates was 2,983 million or on average 497 million per plate. The proportion of reads with a correct barcode and restriction enzyme cut site varied from 69% to 83% per plate with an average of 76%. Alignment to the *S. nigrocinctus* reference genome resulted in 79% overall alignment rate, with the percentage of aligned reads per sample ranging from 56% to 77% (mean = 71%). Filtering of individuals with high percentages of missing genotypes (≥15%) and SNPs with low genotyping rates (≤20%) resulted in the final sample size of 398 individual fish (321 in 2014 and 77 in 2015) and 11,146 SNPs.

**FIGURE 3:**
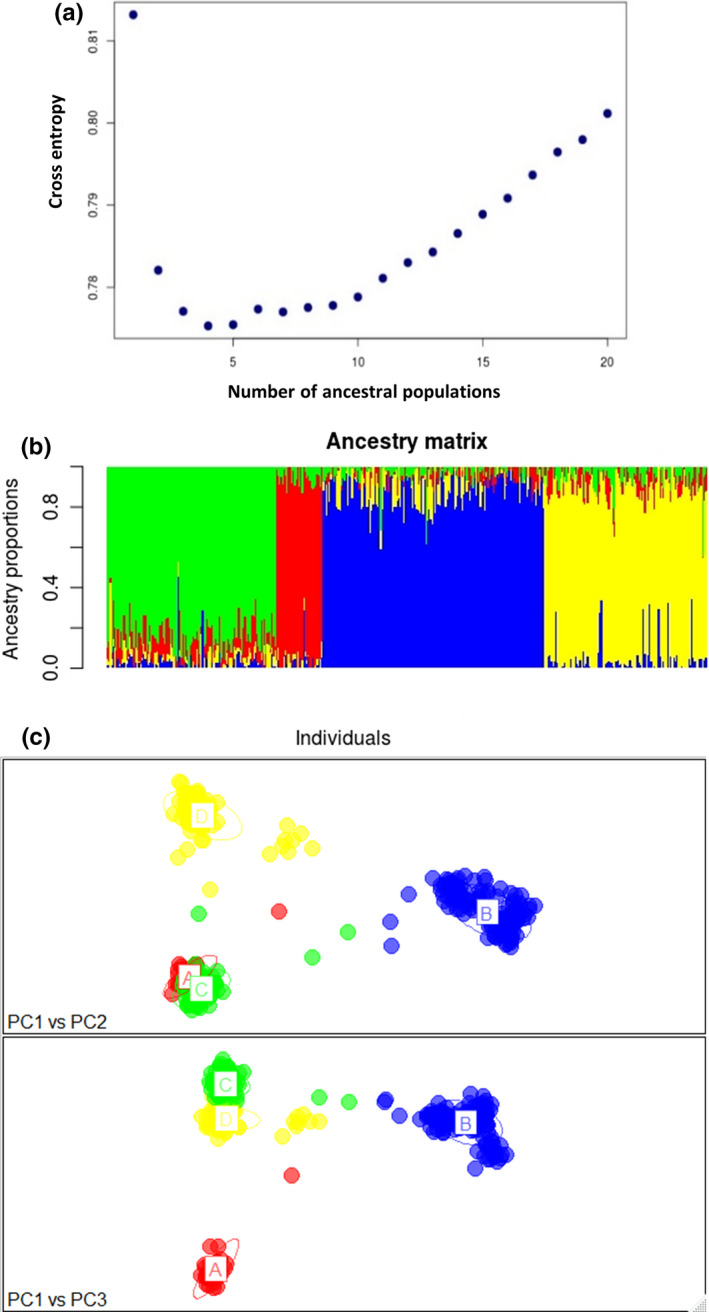
sNMF ancestry analysis revealed 4 ancestral populations represented by fish collected in both 2014 and 2015: (a) cross‐entropy plot for the number of populations in sample; (b) sNMF population ancestry barplot; and (c) PCA analysis with the colors corresponding to the sNMF‐derived majority ancestry populations in panel (b). Note that both years were included concurrently in the analysis. For clarity, year designation is omitted as each population cluster contains both years interspersed throughout

**FIGURE 4:**
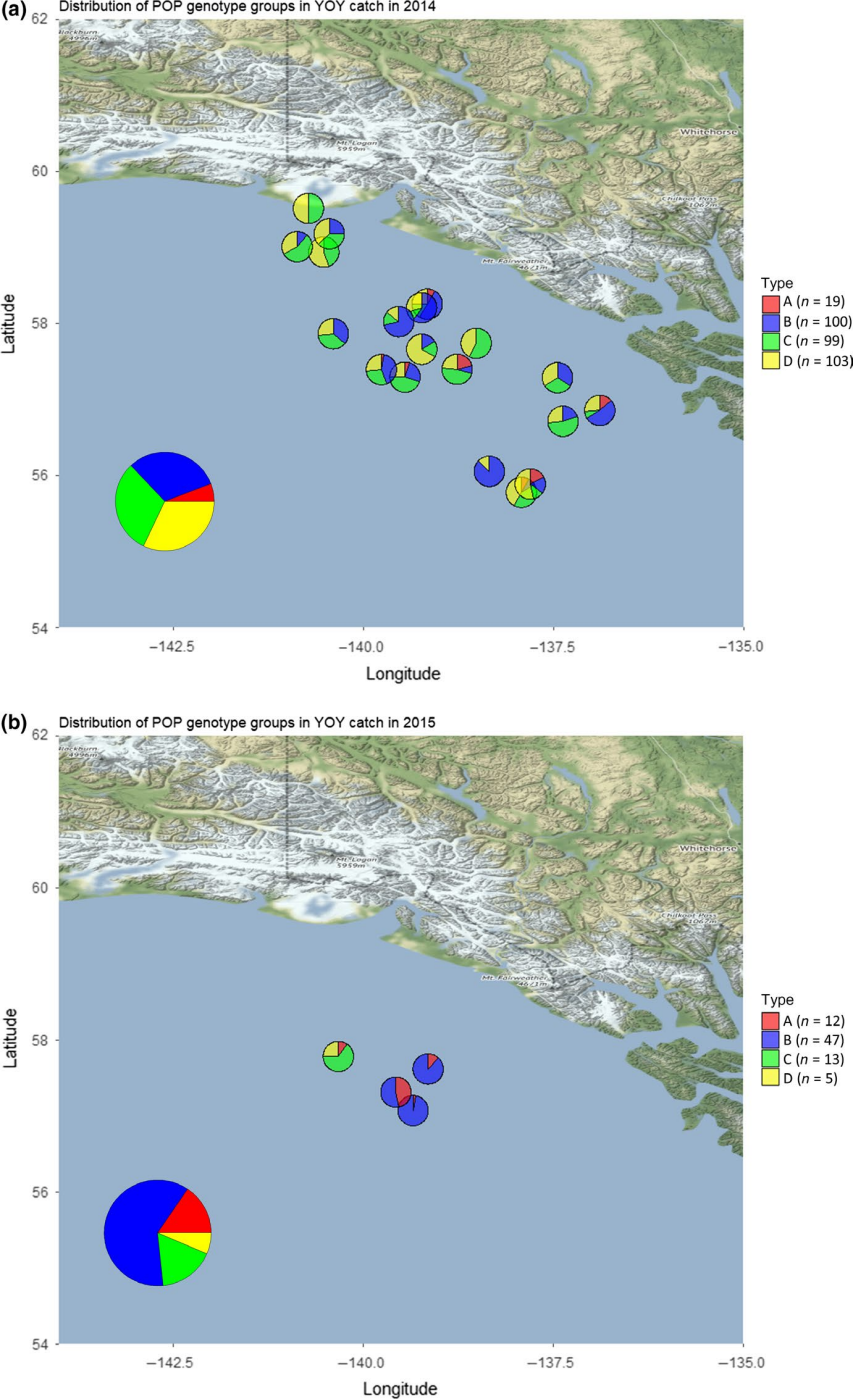
Spatial distribution and the proportional representation of the putative populations in the samples in 2014 (a) and 2015 (b) collections

The ancestry analysis revealed the presence of 4 discrete spawning populations. sNMF ancestry analysis in LEA revealed 4 populations based on cross‐entropy criteria (Figure 3a,b). PCA analysis supported the *K* = 4 sNMF‐derived putative population clusters (Figure 3c). STRUCTURE (Pritchard, Stephens, & Donnelly, 2000) analysis also supported *K* = 4 populations, but with greater admixture of population A and C that was estimated via sNMF algorithm. All four of these populations were represented in both the 2014 and 2015 collections (Figure 4). We also examined whether the population assignments were due to library preparation or sequencing artifacts, and found that all four putative populations were represented in all six 96‐well plates.

Pairwise *F*_ST_ values among putative populations across both years ranged from 0.008 to 0.031 with the biggest differences being from population D (D:C = 0.031, D:B = 0.024, D:A = 0.024). The apparent discrepancy between the pairwise *F*_ST_ distances and the PCA representation (Figure 3c) is due to the imbalance in the number of observations (*n*_A_ = 31, *n*_B_ = 147, *n*_C_ = 112, *n*_D_ = 108) (McVean, 2009). Subsampling (without replacement) equal sample sizes from each population grouping revealed large changes in PCA representation, but little relative change in pairwise *F*_ST_ values (Figure S1). Pairwise *F*_ST_ values for genetic differentiation among putative population–year combinations revealed consistent differentiation between populations in each year (Table 1). Additionally, this difference was conserved across years, meaning little differentiation as measured by *F*_ST_ was observed within a population, between years. These findings support the results of the ancestry analysis and provide evidence that the 2014 and 2015 collections are composed of similar mixtures of discrete spawning populations.

**TABLE 1:** Pairwise *F*_ST_ values (*F*_ST_ below diagonal and *p*‐value above the diagonal) between sNMF‐derived populations and sampling year. There is little genetic differentiation as indicated by *F*_ST_ within a population between years (bolded F_ST_ and corresponding *p*‐values).

	2014‐A	2014‐B	2014‐C	2014‐D	2015‐A	2015‐B	2015‐C	2015‐D
2014‐A	*	.001	.001	.001	**.484**	.001	.001	.001
2014‐B	0.030	*	.001	.001	.001	**.021**	.001	.001
2014‐C	0.012	0.023	*	.001	.001	.001	**.303**	.011
2014‐D	0.014	0.023	0.009	*	.001	.001	.001	**.329**
2015‐A	**0.000**	0.030	0.011	0.014	*	.001	.001	.001
2015‐B	0.032	**0.001**	0.026	0.026	0.032	*	.001	.001
2015‐C	0.011	0.023	**0.000**	0.009	0.012	0.026	*	.017
2015‐D	0.013	0.023	0.007	**0.000**	0.013	0.026	0.008	*

Relatedness analysis showed no related individuals (up to half‐siblings) in the collections. This indicates that the discrete sNMF‐derived populations are not simply groups of closely related individuals. Furthermore, the results of this analysis ensured that no related individuals are included in the subsequent genotype–environment association models, which is thought to result in higher false‐positive rates due to lack of independence among the samples (Newman, Abney, McPeek, Ober, & Cox, 2001; Voight & Pritchard, 2005).

Fewer private alleles were detected in each putative population in 2015 than in 2014, and this pattern was significant when adjusting for the smaller sample size in 2015 (Table 2). This analysis was done separately for each sNMF‐derived population, and we detected private alleles in common among all four populations (Table 3) indicating the same suite of alleles was not detected in 2015.

**TABLE 2:** Number of private alleles in the given year specific to each sNMF‐derived population. Private alleles are the number of alleles that were detected in only one year. For example, 127 is the number of alleles detected in population A in 2014, but not in 2015.

Population	Sample size		Private alleles	
	2014	2015	2014	2015
A	100	47	127^a^	1^b^
B	103	5	2,101^a^	0 ^b^
C	19	12	604	149
D	99	13	401	4

^a^ Significantly more private alleles in 2014 than expected by chance.

^b^ Significantly fewer private alleles in 2015 than expected by chance.

**TABLE 3:** Number of private alleles in common between the sNMF‐derived populations that were detected in 2014 samples but not in 2015. For example, 30 is the number of alleles detected in 2014 in both populations A and B that were not detected in 2015 in either A or B population.

Population	A	B	C
B	30		
C	4	181	
D	5	180	74

### 3.2 Genotype–environment association

The results of LFMM analysis linking environmental and phenotypic variables to SNP variants indicated similar patterns of association with latitude and collection date in both years (Table 4). Of the 76 SNPs associated with these variables in 2014 and 305 in 2015, ten were shared between years (Table S1). The loci common in both years were significantly associated with latitude and collection date only. However, because of the sampling being conducted in a generally south to north direction, sampling date and latitude are collinear. This may possibly indicate a temporal gradient of selection where less fit individuals, those with deleterious alleles, die off during their first months of life. Therefore, the fish collected at later dates may be a subset of the fitter individuals as compared to earlier collections. Or there may be a true latitudinal gradient, or a combination of both factors contributing in various proportions to a selection gradient. Chlorophyll concentration and seawater temperature did not appear to influence loci in 2014, but were associated with 100 loci in 2015.

**TABLE 4:** Results of LFMM analysis and the number of putative loci under divergent selection in 2014 and 2015. The sampling date and latitude (both are confounded as sampling was generally in the northward direction) were consistently associated with selection pressure in both years. However, other environmental factors only showed signatures of selection in 2015.

Gradient	Selected loci	
	2014	2015
Latitude	14	56
Sampling date	62	101
Temperature	0	16
Chlorophyll	0	100
% Lipid	0	27
Condition Index	0	5

**FIGURE 5:**
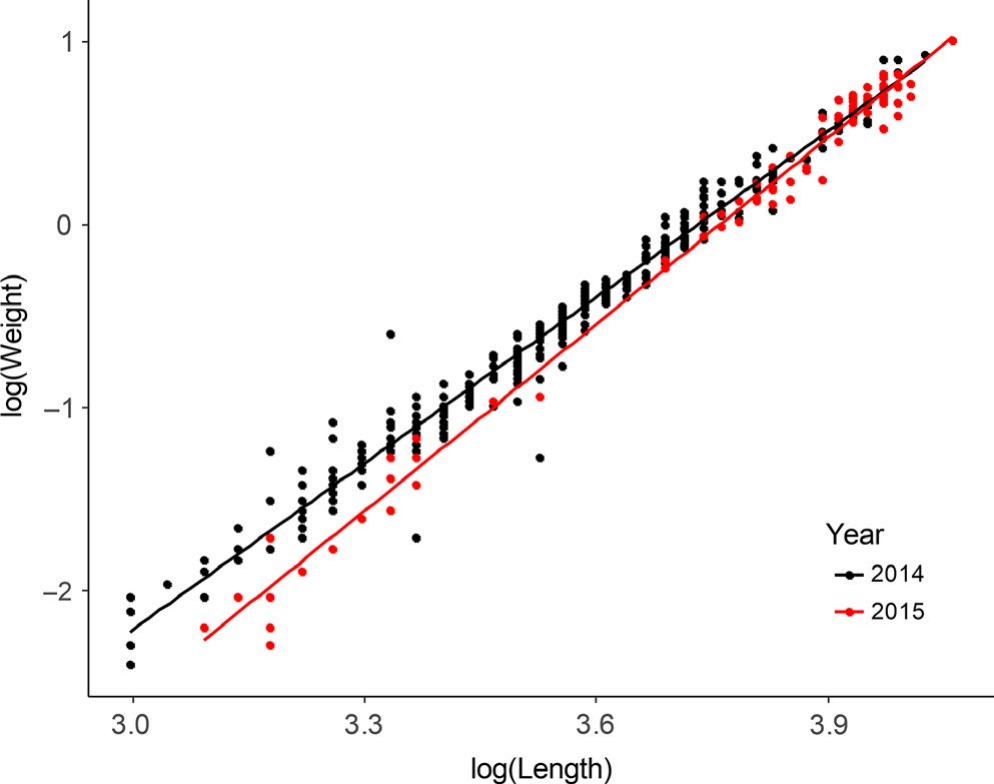
Relationships of body length and weight in the 2014 (black) and 2015 (red) young‐of‐the‐year Pacific ocean perch. In 2015, smaller length fish had significantly (*p* < .0001) smaller mass than in 2014, indicating environmental factors in 2015 may have negatively influenced their condition.

In 2015, the fish experienced poorer growing condition as compared to 2014 (Cavole et al., 2016). This was evident in their weight for a given length when examining the condition index graphs (Figure 5). Linear regression analysis indicated a significantly (*p* < .05) lower intercept and steeper slope in 2015 suggesting that smaller fish had poorer condition in 2015, but larger fish appeared to be unaffected. Whether the smaller fish died off and only larger fish survived is uncertain, although there appears to be a genetic basis of selection where a number of loci were identified as being associated with fish body condition (% lipid and condition index). This was not observed for the fish collected in 2014.

### 3.3 Gene ontology enrichment

BLAST search resulted in only six loci being associated with known genes in 2014 and 24 in 2015 (Table S2). The broad‐scale biological processes associated with the gene ontology (GO) enrichment are listed in Table S3, while detailed information and fine‐scale biological processes associated with the gene subsets may be found in the supplementary materials. The majority of genes were associated with developmental processes: 4 out 5 in 2014 and 108 out of 168 in 2015 (Table S3). Intracellular processes were associated with all environmental gradients in both years (see Appendix S1). Various developmental processes were mostly associated with collection date, chlorophyll‐a concentration, latitude, water temperature, and tissue lipid percentage. Growth‐associated processes were mostly associated with chlorophyll‐a concentration, collection date, collection latitude, water temperature, and tissue lipid percent, but not condition index. Metabolic‐related processes were associated with condition index, latitude, and temperature. Fatty acid and lipid metabolism processes were only associated with a temperature gradient. This functional analysis revealed the expected patterns of selection on ontogenic biological processes for these young‐of‐the‐year fish, and we did not look further at SNPs located in introns for nearby genes.

## 4 DISCUSSION

### 4.1 Sympatry and population structure

One of the surprising findings of this study was the strong genetic clustering where the individuals of respective clusters were dispersed among the sampling locations, as well as conserved between the two years (Figure 4). This is consistent with the predictions from the DisMELS model (Stockhausen, 2009), but somewhat in contrast with the findings of Kamin et al. (2014), where the collections were mapped to the closest adult groups and no genetic clustering was detected except for interannual divergence at sampling locations, indicating possible difference in population mixtures. However, their study only used fourteen microsatellite markers with frequencies at each sampling locations for dozens of individuals and therefore may have lacked statistical power to detect the finer‐scale genetic clustering compared to the RADseq approach we employed here. We examined the first three principal component loadings to determine whether certain regions of the genome had greater influence on the separation of the four clusters, but found the most influential loci distributed throughout (Figure S2). Also, the Kamin et al. (2014) study treated each haul collection as a sampling unit and conducted tests on the allele frequencies among the hauls, transects, locations, and years, but did not examine genetic clustering based on individual admixture analysis. However, the presence of genetic structure in our study is consistent with Palof et al. (2011), who detected isolation‐by‐distance population structure in the adults. This inference is supported by the similar *F*_ST_ values detected here. We calculated *F*_ST_ ranging from 0.008 to 0.032 between clusters, and similarly, Palof et al. (2011) calculated *F*_ST_ values between <0.01 and >0.03. This indicates similar levels of differentiations among the sNMF‐derived populations and observed distinct adult populations. The complete mixing among the genetically distinct groups of YOYs would be expected to result in a lack of population structure within just a few generations if the mixed fish maintained their grouping through settlement, recruitment, and spawning. Our observations are consistent with both the DisMELS (Stockhausen, 2009) and Palof et al. (2011) results indicating dispersal may not be the primary mechanism by which POP population structure is maintained suggesting some level of larval retention is needed to maintain it.

Our study suggests that distinct Pacific ocean perch populations that are sympatric during the larval and YOY stage are likely geographically segregated and genetically differentiated during spawning. The presence of genetic clusters in spite of larval stage sympatry may indicate that once the fish settle out in the nearshore rearing habitat, they may be able to home‐in to their natal locations over the following few years. If homing to their natal locations begins after fish settle out of the water column into their nearshore rearing habitat, then the mixtures of genotypes would be evident among larvae as they advected toward shore by cross‐shelf currents.

The homing behavior in adult *Sebastes* spp. has been well documented (i.e. Carlson & Haight, 1972; Carlson, Haight, & Helle, 1995; Matthews, 1990). It is unknown, however, when this behavior begins. Schools of age 1+ fish are spatially segregated (Carlson & Haight, 1976), although it is unknown whether those individuals are from a single or multiple source populations. It may be that these single‐cohort schools are composed of individuals from multiple sourced populations and like salmon, leave the school when natal location is nearby.

Homing behavior would result in genetic isolation and population structure consistent with our observations. Westrheim (1975) noted that POP schools were separated by bathymetry and would not cross deep trenches once in demersal stage. Withler, Beacham, Schulze, Richards, and Miller (2001) also described POP populations that were genetically distinct, yet lived within close proximity of each other, even when sampled in different seasons. Therefore, if larvae from discrete nearby parturition locations, separated by bathymetric features such as canyons and ridges, were jointly entrapped in the oceanic currents, these clusters would resemble our observations. If homing to their natal locations begins after fish settle out of the water column into their nearshore rearing habitat, then the mixtures of genotypes would be evident among larvae as they advected toward shore by cross‐shelf currents.

Another explanation for the fate of these YOY fish is that they are entrained in the coastal current and mesoscale eddies and fail to find suitable rearing habitat prior to winter settlement and are therefore destined to die, and our sampled fish were already the “swimming dead.” The selection that we observed would then be the sign of various phenotypes dying at different rates, while the unobserved fish, the ones that did not get advected away from natal grounds and mixed with other similar‐fated YOYs, are the only ones that successfully reach suitable nearby rearing habitat. Westrheim (1958), and Carlson and Haight (1976) noted the extreme successes and failures among POP year classes, which perhaps may be indicative of different advection ratesaway from the natal grounds or high larval mortality, assuming consistent spawning population.

### 4.2 Genome–environment association

Fish employing broadcast spawning strategies characterized by larval and juvenile pelagic drift in ocean currents are subject to large interannual variability in oceanic conditions (Stockhausen et al., 2018). Stockhausen et al. (2018) refer to this as “running the gauntlet,” as it is during this critical life stage that these fish are most vulnerable, experiencing the highest rates of mortality. This vulnerability is not only due to the vagaries of physical transport, but also due to their physiological condition where they must meet energetic demands of acquiring sufficient lipid reserves in order to move to inshore nursery areas.

During years of favorable ocean conditions with ample food availability, such as 2014 for POP, mortality may be low and selection weak, allowing most phenotypes to survive through the pelagic phase and into nearshore settlement. However, during years of unfavorable ocean conditions, such as the unusual warming, low primary productivity, and low food availability in 2015 for POP, mortality may be high. If this increase in mortality is especially high for certain phenotypes, the selection may be strong, with only the most favorable phenotypes surviving to settlement.

Our results show consistent selective forces along the sampling date/latitude gradient in both 2014 and 2015 for POP with 10 of the 381 putative selective loci being in common in both years (Table 4). The LFMM analysis was done independently for each of the years, and finding the same putative selected loci in both years is surprising. And although the LFMM method purportedly accounts for demographic factors such as population mixtures, the date/latitude gradient association could be due to adult spawning populations being differentiated at these loci. Based on timing and location of spawning, their progeny may follow the spatiotemporal pattern identified by GEA. This is further supported by the distribution of the sNMF identified genetic clusters in relation to their distribution as seen in Figure 4. Alternatively, this may indicate that the spawning adult populations contain a high proportion of alleles at those loci that in 2014 and 2015 years were deleterious to the YOY progeny encountering the environmental conditions during their pelagic developmental stage. Since POP are very long‐lived and may even spawn into their 100th year (Conrath & Knoth, 2013; Heppell, Heppell, Spencer, Smith, & Arnold, 2010; Hulson et al., 2017), some of the alleles in the parental population are expected to have been selectively advantageous during their respective first year at sea; therefore, the alleles that were advantageous when the parents were YOY may be deleterious in some oceanic conditions encountered by their progeny decades later. It is then expected that patterns of selection as displayed by the subsets of selected alleles would be cohort‐specific.

In both years, at each sampling location, we found an apparently random distribution of sNMF‐derived populations, and within each population, there was considerable overlap in the specific private alleles (Table 3). We would not expect all the populations to experience similar gene flow or other demographic processes and attribute this to selection pressure that was jointly experienced by all individuals. This is consistent with their biology and high mortality experienced from parturition to settlement. Interannual differences in the strength of selection pressure were evident when comparing the 2014 and 2015 sample sizes. The sampling effort was similar in both years, but the paucity of all forage fish in 2015 as compared to 2014 was striking. In this r‐selected species, these large die‐offs are indicative of unfavorable environmental conditions creating widespread mortalities due to strong selection pressures. Furthermore, due to the larger sample size in 2014 (321) than in 2015 (77), we would expect more putative selected loci in 2014 just due to the increase in statistical power, but that was not the case. In 2014, the oceanic conditions were typical (Cavole et al., 2016), with large YOY abundances in the ocean, and no putative selected loci were identified aside from those associated with collection date/latitude. However, in 2015, the oceanic conditions were abnormal with warmer sea surface temperatures (Gentemann et al., 2017) and were marked by large seabird die‐off (Jones et al., 2018). This likely resulted in stronger selective pressure on YOY in 2015, and this is supported by the greater number of putative selected alleles. Therefore, by the time the 2014 and 2015 cohorts settled out in the nearshore, we expect that most individuals have gene variants that were most favorable and selected for by the conditions encountered in that year.

The difference in the change in condition index indicates different growth conditions between the two years. In 2015, the smaller fish had less mass than in 2014, but the larger fish had equivalent mass in both years (Figure 5). This indicates that in 2015, a much warmer year than in 2014, the smaller fish were unable to gain weight as compared to the same sized fish in 2014. If the temperatures were still within optima for POP YOY growth, then smaller size suggests smaller‐sized prey items were either unavailable or of insufficient nutritional value to support the higher growth rates predicted by the higher temperature in 2015. However the larger fish in both years were equally successful at gaining mass. This suggests that the environment in 2015 imposed a larger variance in fitness and therefore much stronger selection pressure, and this is consistent with the greater number of putative selected loci in 2015 than in 2014. This is further supported by the recruitment estimates in the 2017 stock assessment with 2014 cohort being 87.5 million and 38.2 million in 2015 (Hulson et al., 2017).

### 4.3 Gene ontology enrichment

The GO enrichment analysis yielded particularly interesting and intuitive results. The selective processes identified here act during the developmental and high growth larval life stage, and 87% of the general biological processes associated with the LFMM identified putative selected genes directly corresponded to development and growth. The remaining 7% and 5% were associated with intracellular processes and metabolism, respectively. Furthermore, in 2014, we did not identify any biological processes associated with growth or metabolism, indicating that the early life conditions were favorable across the habitat surveyed, with little selection acting on those gene variants. The numerous processes identified in 2015, however, may be indicative of unfavorable oceanic conditions, leading to a significant loss of phenotypes with the deleterious gene variants. Because 2015 was an unusually warm year (Gentemann et al., 2017), it is not surprising that these warmer temperatures would directly affect metabolic processes. This is underscored by our finding of associations between fatty acid, lipid metabolism, and temperature for 2015, but not for 2014.

### 4.4 Fluctuating selection and maintenance of adaptive diversity

**FIGURE 6:**
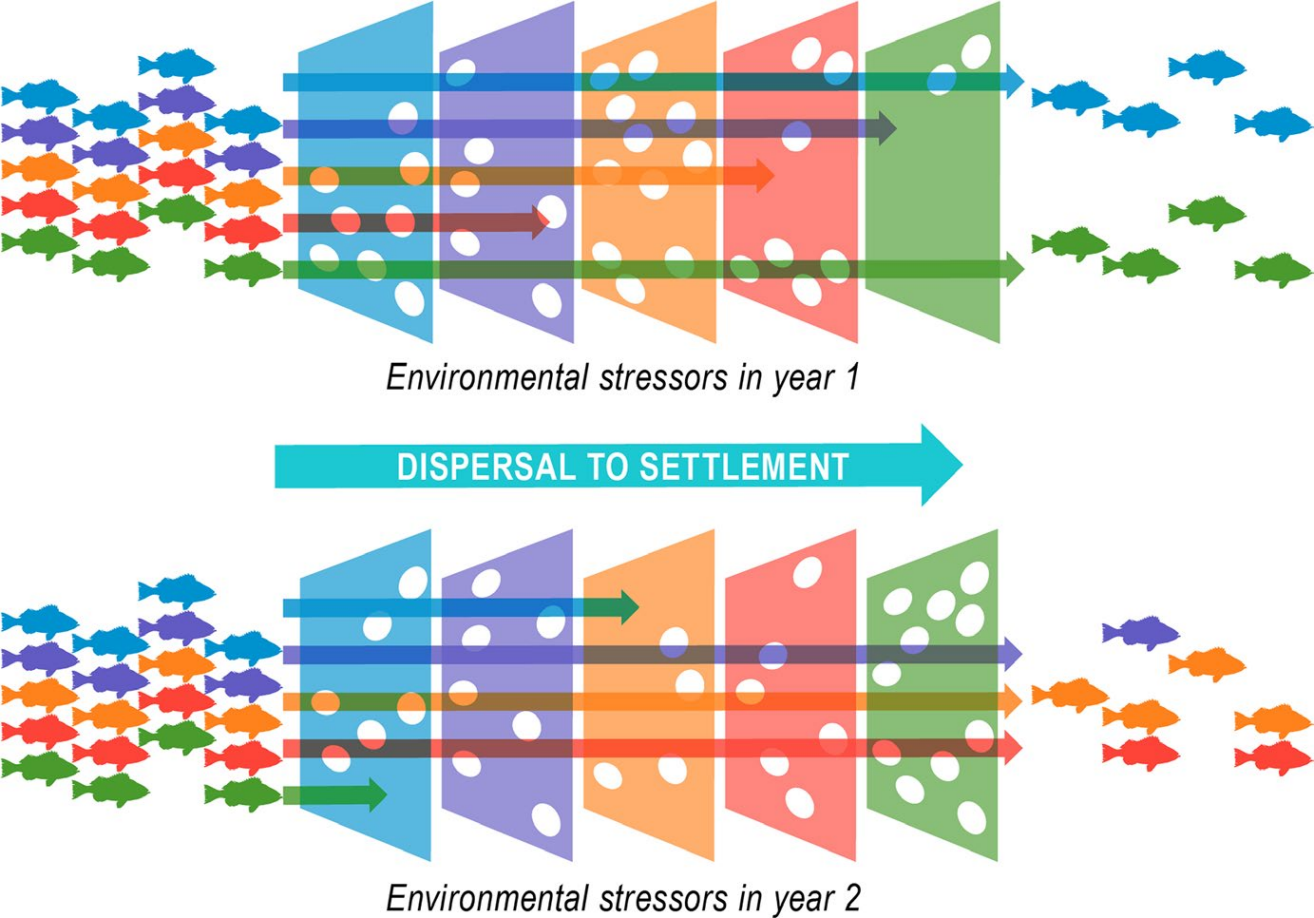
The selective sieve. The five colored plates represent various hypothetical environmental forces (such as temperature and chlorophyll density) that are highly variable among years. This represents the different selection pressures encountered by the Pacific ocean Perch during initial pelagic life stage. Each years' cohort therefore contains the alleles that were selected for during their first year. Populations of long‐lived adults representing multiple cohorts maintain genetic diversity as a result of this temporal variation in selection

Our results suggest the presence of a temporal portfolio effect, where a multiage population with overlapping generations maintains a portfolio of genotypes (Ellner & Hairston, 1994). The interannual variation in oceanic conditions and its effects on the selection of POP during their first year at sea prior to settlement may be thought of as a “Selective Sieve” (Figure 6), where each year represents different sets of selection pressures during the early developmental life stage. The selection pressures, in the form of various environmental drivers such as ocean temperatures, productivity (chlorophyll‐a), and their timing vary from year to year resulting in some phenotypes being detrimental in one year, but advantageous in another when encountering highly diverse pelagic habitats. The selective sieve is therefore specific to the year of the POP pelagic life stage and unique to each cohort which then contains alleles favored by the conditions of their first year. In these long‐lived species with life spans of over 100 years, in any one year the larval cohort at parturition may be the result of breeding across dozens of spawning aged cohorts (~8–100 years old or more). The parental cohorts contain many alleles that are representative of the selection due to oceanic conditions during their first year at sea. At parturition, the POP larvae contain all of these alleles; however, from parturition to settlement, some of the alleles may prove to be deleterious as the oceanic conditions are not favorable resulting in mass die‐offs as evident in r‐selected species. Eventually, only a subset of the larvae containing the advantageous alleles survive until settlement. This is an example of fluctuating selection (Bell, 2010; Kawecki, 2000; Lande, 2007), where the direction of selection is constantly changing between generations. The species’ life history of long reproductive period relative to the time scale of fluctuating selection maintains genetic diversity that is adaptive across a range of environmental variation.

Population viability in fish employing broadcast spawning strategies is especially vulnerable to changing oceanographic conditions. Ocean currents may advect YOY far offshore where they will fail to reach their shelf‐slope nursery areas. Using ROMS‐based models, Stockhausen et al. (2018) showed that up to 70% of the YOY failed to reach suitable nursery habitats prior to wintertime and were not expected to survive. The ones that are not advected out of reach of nursery habitat must still acquire sufficient lipid reserves in order to settle out and overwinter. Interannual differences in ocean temperatures, prey and predator abundances and composition will also affect whether the YOY will survive to reach their nursery habitats with sufficient lipid reserves to overwinter and eventually recruit to the population. Maintaining a high diversity in phenotypes through cohort‐specific selection may be thought of as a form of diversification bet‐hedging response to a fluctuating natural selection as described by Simons (2009).

These results underscore the importance of maintaining many cohorts in order to maximize the population resilience to environmental variability. POP are vulnerable to age truncation where older fish are more likely to be fished since they are exposed to fishing longer (Berkeley, Hixon, Larson, & Love, 2004). The importance of maintaining older age classes in marine fishes has long been recognized as being a factor in their recruitment (Hixon, Johnson, & Sogard, 2013; Longhurst, 2002). Hanselman, Heifetz, Fujioka, and Ianelli (2005) noted that age truncation has occurred in POP due to unrestricted fishing in the past which led to disproportional absence of 40+ year old fish. However, the mechanism of adaptation through maintenance of age‐specific advantageous alleles would be compromised if whole cohorts are inadvertently fished by depriving populations of the advantageous alleles specific to that cohort.

The uniqueness of the demonstrated cohort‐specific selection signatures may allow for reconstruction of past oceanographic conditions based on the alleles present in a given cohort. The 2015 cohort will therefore represent the alleles favored (or conversely lost) during especially warm oceanic conditions as experienced during 2015. It may be possible that by examining allele frequencies in an adult cohort, of for example 50‐year‐old fish, the selection pressures encountered during their YOY stage may be revealed. Furthermore, aging of adults based on cohort‐specific allelic signatures may also be possible by maintaining cohort‐specific selected allelic signatures. This may prove especially useful since otolith aging of POP adults is fairly error‐prone especially for older fish (>20 years old) (Beamish, 1979; Stanley, 1986).

## 5 CONCLUSIONS

We found evidence for different selective pressures for Pacific ocean perch YOY across two years that had very different environmental conditions. These results provide evidence that long‐lived marine species such as POP may be resilient to natural environmental variability by maintaining a portfolio of adaptive alleles resulting from selection encountered by each cohort during their most vulnerable life stage from parturition to settlement. However, this resilience may be limited to the environmental conditions that prevailed in the last few centuries. The “selective sieve” framework may provide valuable insights into other species employing similar life history strategies. Hoffmann and Sgro (2011) note that species facing strong but fluctuating selection pressures, such as YOY POP during the pelagic life stage, will have a difficult time adapting. Here, we demonstrated an exception where due to the way POP are able to maintain these selected alleles may allow them to be especially adaptable under fluctuating environmental conditions. Pacific ocean perch life history of dozens of cohorts spawning to produce a new cohort presents an almost ideal system to test the portfolio effect where the high genetic diversity in parents is conserved for multiple adjacent years resulting in similar allele frequencies between closely aged cohorts. By examining relative strengths of selection among discrete populations and adult cohorts, we are able to jointly examine spatial and temporal portfolio effects. However, we need to underscore that the strong signatures of fluctuating selection we observed may in fact be the result of the interannual sampling difference or other nonadaptive evolutionary forces. This may be especially pronounced due to the large difference in the sample sizes between the two years. In the future, we plan to sample across adult populations to link genetic variation to larval cohorts and adult habitat/geographic population structure.

## CONFLICT OF INTEREST

None declared.

## AUTHOR CONTRIBUTION

**Jacek Maselko:** Conceptualization (lead); Data curation (lead); Formal analysis (lead); Funding acquisition (lead); Investigation (lead); Methodology (lead); Project administration (equal); Resources (equal); Software (equal); Writing‐original draft (lead); Writing‐review & editing (equal). **Kimberly R. Andrews:** Conceptualization (supporting); Data curation (supporting); Formal analysis (supporting); Methodology (supporting); Project administration (equal); Writing‐review & editing (equal). **Paul A. Hohenlohe:** Conceptualization (supporting); Data curation (supporting); Formal analysis (supporting); Investigation (supporting); Methodology (equal); Project administration (lead); Writing‐review & editing (equal).

## Supporting information

Appendix S1

## Data Availability

Sequence data and sample information are available on Dryad https://doi.org/10.5061/dryad.d7wm37pz7
