# Supplementary material for: Long‐lived marine species may be resilient to environmental variability through a temporal portfolio effect
Source: Ecol Evol. 2020 May 25;10(13):6435–48. doi: 10.1002/ece3.6378 (PMC7381576; doi:10.1002/ece3.6378)
Supplement: Supplementary file 1 — Appendix S1 [file ECE3-10-6435-s001.docx]

**Supplementary Materials**


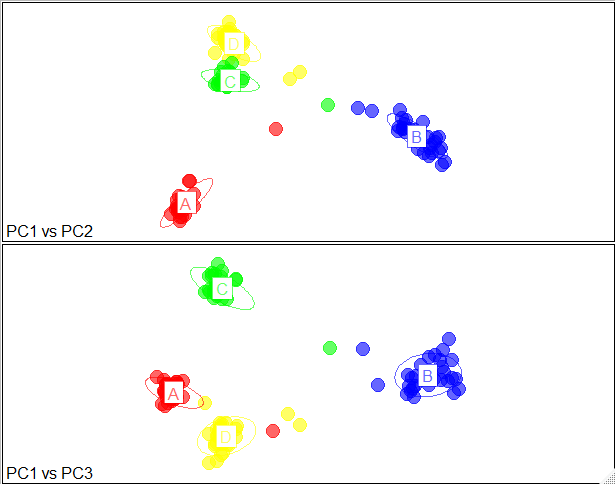

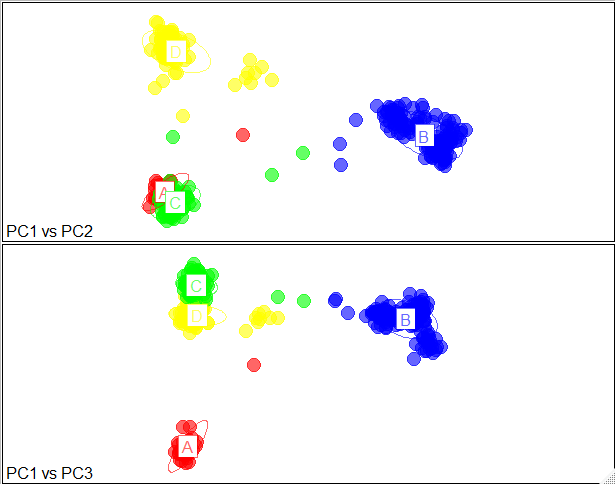


(a)

(b)


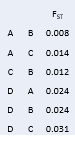

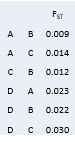


Figure 1. Unlike *F*_ST_ values, which are robust to changes in sample sizes, inference based on PCA representation may lead to erroneous interpretations: (a) PCA plot and *F*_ST_ values (in box) based on original sample sizes (n_A_=31, n_B_=147, n_C_=112, n_D_=108); (b) PCA plot and *F*_ST_ values (in box) based on subsampled without replacement equal sample sizes of n_A_=n_B_=n_C_=n_D_=30. Note little change in pairwise *F*_ST_ values, but large changes in PCA representation of population distances due to differences in relative sample sizes among groups (McVean 2009).


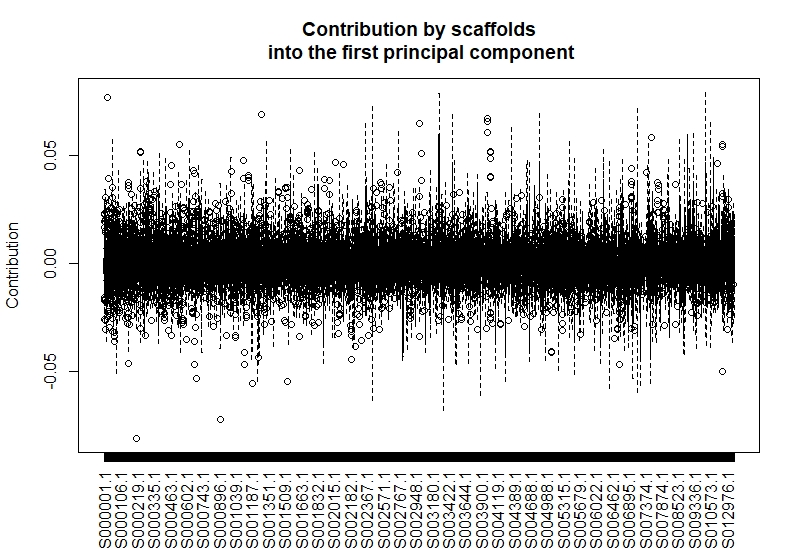


Figure 2. The loadings plot of the contribution of the loci at each scaffold in the first principal component to the overall cluster differentiation.

Table 1. Putative selected loci and their associated environmental gradient that were in common in both 2014 and 2015 years.

|  | | | **Environmental Gradient** | | |
| --- | --- | --- | --- | --- | --- |
| **Locus** | **Year** | **Date** | **Latitude** | **Chlorophyll** | **% Lipid** |
| **10101** | 2014 | X |  |  |  |
|  | 2015 |  | X |  |  |
| **2088** | 2014 | X |  |  |  |
|  | 2015 | X | X | X | X |
| **24006** | 2014 |  | X |  |  |
|  | 2015 | X |  | X |  |
| **25342** | 2014 | X |  |  |  |
|  | 2015 | X |  | X |  |
| **2579** | 2014 | X |  |  |  |
|  | 2015 |  | X | X |  |
| **28846** | 2014 | X | X |  |  |
|  | 2015 | X | X | X | X |
| **29782** | 2014 | X | X |  |  |
|  | 2015 | X | X | X |  |
| **343** | 2014 | X |  |  |  |
|  | 2015 | X |  | X |  |
| **8626** | 2014 | X |  |  |  |
|  | 2015 | X | X | X |  |
| **922** | 2014 | X | X |  |  |
|  | 2015 | X |  | X |  |

Table 2. List of BLAST matches for the selected loci from LFMM in 2014 and 2015.

| **2014** | **Date** | **Lat** |
| --- | --- | --- |
| biogenesis of lysosome-related organelles complex 1 subunit 4-like (BLOC-1) | X |  |
| lysine-specific demethylase 5B-B-like, transcript variant X4 (KDM5B) | X |  |
| neurochondrin (NCDN) | X |  |
| GREB1-like protein | X |  |
| kelch-like protein 36 (KLHL36) | X |  |
| WD repeat domain 3 (WDR3) | X | X |

| **2015** | **Date** | **Lat** | **Temp** | **Lipid** | **Chl** | **Condition Index** |
| --- | --- | --- | --- | --- | --- | --- |
| nucleoprotein (TPR) | X | X | X | X | X | X |
| T-box 20 (tbx20) | X | X | X | X | X |  |
| NGFI-A binding protein 1 (nab1) | X | X |  | X | X | X |
| aldehyde reductase (AKR1A1) gene | X | X |  | X | X |  |
| zinc finger CCCH-type containing 3 (zc3h3) | X | X |  | X | X |  |
| teneurin-1-like (TENM1) | X | X |  |  | X |  |
| retromer complex component B (vps26b) | X | X |  |  | X |  |
| STE20-like serine/threonine-protein kinase-like (SLK) | X | X |  |  | X |  |
| teneurin transmembrane protein 1 (tenm1) | X | X |  |  | X |  |
| alsin-like (ALS2) | X |  |  | X | X |  |
| keratin, type II cytoskeletal 8-like (KRT8) | X |  |  |  | X |  |
| MDS1 and EVI1 complex locus (mecom) | X |  |  |  | X |  |
| ubiquitin specific peptidase 32 (usp32), transcript variant X7 | X |  |  |  | X |  |
| erb-b2 receptor tyrosine kinase 4 (erbb4) | X |  |  |  |  |  |
| collagen type IV alpha 2 chain (col4a2) | X |  |  |  |  |  |
| solute carrier family 25 member 46 (slc25a46) | X |  |  |  |  |  |
| DnaJ heat shock protein family (Hsp40) member A2 |  | X | X | X | X |  |
| enkurin, TRPC channel interacting protein (enkur) |  | X |  |  |  |  |
| myotubularin-related protein 13-like (sbf2) |  | X |  |  |  |  |
| voltage-dependent L-type calcium channel subunit alpha-1D (CACNA1D) |  | X |  |  |  |  |
| putative helicase mov-10-B.2 (MOV-10) |  | X |  |  |  |  |
| phosphoribosyltransferase domain-containing protein 1 (PRTFDC1) |  | X |  |  |  |  |
| trypsin domain containing 1 (tysnd1) |  |  | X |  |  |  |
| alanyl-tRNA synthetase (aars) |  |  |  |  |  | X |

Table 3. Summary of gene ontology and enrichment analysis showing broad biological processes associated with selected genes from LFMM analysis. The numbers in the table indicate how many distinct biological functions were found in each category and do not indicate significance or importance of the associated putatively selected alleles. “Total processes” indicates the total number of distinct biological processes identified, whereas “Discrete processes” indicates the number of broad biological process groups.

| **Broad Biological Process** | **2014** | **2015** | | | | | |  |
| --- | --- | --- | --- | --- | --- | --- | --- | --- |
|  | **Date** | **Chl** | **Date** | **Condition** | **Latitude** | **Lipid %** | **Temp** | **Total** |
| intracellular processes | 1 | 39 | 38 | 22 | 72 | 29 | 12 | 213 |
| **Development** |  |  |  |  |  |  |  |  |
| cell development |  | 6 | 5 | 2 | 2 | 1 | 1 | 17 |
| cell adhesion |  | 1 | 1 |  | 1 |  |  | 3 |
| extracellular matrix organization |  |  | 1 |  |  |  |  | 1 |
| determination of symmetry |  |  |  |  |  | 2 | 2 | 4 |
| embryo development |  | 8 | 6 | 1 |  | 3 | 3 | 21 |
| developmental growth |  | 3 | 6 |  | 1 | 6 | 6 | 22 |
| anatomical structure/organ development | 1 | 2 | 2 |  |  |  |  | 5 |
| epithelium development |  | 2 | 2 |  |  | 2 | 2 | 8 |
| cardiac development |  | 13 | 11 |  | 13 | 16 | 16 | 69 |
| vasculogenesis |  | 2 | 2 |  | 1 | 4 | 4 | 13 |
| liver development |  | 1 | 1 |  | 1 | 1 | 1 | 5 |
| renal system development | 1 | 20 | 19 |  |  | 1 | 1 | 42 |
| digestive system development |  | 4 | 4 |  | 4 | 4 | 4 | 20 |
| gland development | 2 |  |  |  |  |  |  | 2 |
| brain development | 2 |  |  |  |  |  |  | 2 |
| neuron generation |  |  | 4 |  |  |  |  | 4 |
| immune system development |  |  |  |  |  | 1 | 1 | 2 |
| pigment biosynthesis |  |  |  |  | 2 |  |  | 2 |
| **Growth** |  |  |  |  |  |  |  |  |
| non-developmental growth |  |  |  |  |  | 1 | 1 | 2 |
| muscle formation |  | 5 | 5 |  | 5 | 7 | 7 | 29 |
| growth regulation |  | 1 | 1 |  | 1 | 1 | 1 | 5 |
| organ growth |  | 2 | 2 |  | 2 | 2 | 2 | 10 |
| cell growth |  | 3 | 3 |  |  | 1 | 1 | 8 |
| blood production |  |  |  |  |  | 1 | 1 | 2 |
| **Metabolism** |  |  |  |  |  |  |  |  |
| metabolic processes |  |  |  | 13 | 15 |  | 8 | 36 |
| fatty acid metabolism |  |  |  |  |  |  | 3 | 3 |
| lipid metabolism |  |  |  |  |  |  | 2 | 2 |
| Total processes | 7 | 112 | 113 | 38 | 120 | 83 | 77 | 550 |
| Discrete processes | 5 | 16 | 18 | 4 | 13 | 18 | 21 |  |

Algorithm 1. Algorithm to calculate the significance (p-value) of whether fewer private alleles in smaller sample size are by chance or other population forces (i.e. selection).

Let $x_{1}$ and $x_{2}$ be two independent samples drawn from one $\left( F \right)$ or two $\left( G, H \right)$ probability distributions. In the null hypothesis we assume that both samples are drawn from a single distribution $\left( F, H_{o}:G=H \right)$. The test statistic $\hat{a}_{Li}^{*}$ is the number of alleles lost when we sample n_i_ samples without replacement from the F distribution. This procedure is permutated B times (i.e. B=1,000) and the p value calculated as ${Prob(\hat{a}}_{Li}^{*}\geq\hat{a}_{Li})$ the probability of losing at least $\hat{a}_{Li}$ alleles by chance given sample size n_i_. Note that the probability of losing less than or equal to $\hat{a}_{Li}, {Prob(\hat{a}}_{Li}^{*}\leq\hat{a}_{Li})$ is then: $1-{Prob(\hat{a}}_{Li}^{*}\geq\hat{a}_{Li})$.

**Usage**

Pvalue_private_alleles(x.1,x.2, nperm)

**Arguments**

**x.1** n by a data frame of first sample genotypes where a are alleles

**x.2** m by a data frame of second sample genotypes where a are alleles

**nperm** number of permutations.

**Value**

Data frame with sample size of 1^st^ sample, number of alleles lost in 1^st^ sample, and P-value for the probability of losing that many alleles by random chance; followed by sample size of 2^nd^ sample, number of alleles lost in 2^nd^ sample, and P-value for the probability of losing that many alleles by random chance;

Pvalue_private_alleles<-function(x.1,x.2, nperm) {

A.1 <- length(S.1[which(colSums(x.1)==0)]) # Number of missing alleles in group 1

A.2 <- length(S.2[which(colSums(x.2)==0)]) # Number of missing alleles in group 2

X <- rbind(x.1,x.2)

N <- nrow(X)

ALoss.1 <- c()

ALoss.2 <- c()

for (b in 1:nperm){

s <- sample(N, nrow(x.1), replace=FALSE)

P.1s <- colSums(X[s,])

P.2s <- colSums(X[-s,])

ALoss.1 <- rbind(ALoss.1,length(P.1s[which(P.1s==0)])) # alleles lost in 1

ALoss.2 <- rbind(ALoss.2,length(P.2s[which(P.2s==0)])) # alleles lost in 2

}

Pval.1 <- length(ALoss.1[ALoss.1>=A.1])/nperm

Pval.2 <- length(ALoss.2[ALoss.2>=A.2])/nperm

result <- data.frame(n1=nrow(x.1),AlleLoss1=A.1, Pvalue1=Pval.1,n2=nrow(x.2),AlleLoss2=A.2, Pvalue2=Pval.2)

return(result)

}
